# A Non-Stationary Model for Analysis of Impedance Spectra of Biological Samples

**DOI:** 10.3390/e28030291

**Published:** 2026-03-04

**Authors:** Gabriela Janik, Urszula Kamińska, Marta Kasprzyk, Leszek Niedzicki, Teodor Buchner

**Affiliations:** 1 Faculty of Physics, Warsaw University of Technology, Koszykowa 75, 00662 Warszawa, Polandteodor.buchner@pw.edu.pl (T.B.); 2 Faculty of Chemistry, Warsaw University of Technology, Noakowskiego 3, 00664 Warszawa, Poland; marta.kasprzyk@pw.edu.pl (M.K.); leszek.niedzicki@pw.edu.pl (L.N.)

**Keywords:** impedance spectrum, biological samples, biopotential, Poisson Boltzmann equation, diffusion equation

## Abstract

Electric impedance spectrum (EIS) is attracting attention in many areas of science, ranging from electrochemistry and material science to medical diagnosis. Interestingly, theoretical description often stops at material constants and specific physical mechanisms are represented by equivalent circuit elements, which is also motivated by the common use of various bridge methods. This specifically applies to biological samples, which exhibit a rich variety of responses to the electric field. Here, we present a step further from the description that utilizes equivalent circuit elements. We demonstrate how alteration of the mesoscopic structure affects the EIS in a biological sample: a cucumber under thermal treatment that comprises a cooling and warming phase. As the freezing temperature of water is exceeded during the cycle, the cucumber becomes frosted, which leads to unrecoverable changes in the internal structure, with no change of chemical composition. The experimental evidence is complemented by theoretical analysis, based on a novel approach to modeling non-stationary problems, derived from the stationary Poisson–Boltzmann equation. We demonstrate a qualitative agreement between the theoretical and the experimental results, and discuss the procedure for tuning the model. We also demonstrate that, of the temperature variations of the position of the beta dispersion, the one related to the mesoscopic structure, can be used to assess the ionic strength of the material, determine the microscopic diffusion constant, or reflect the changes in mesoscopic structure, depending on experimental protocol.

## 1. Introduction

Electric impedance is a technique that seems to be gaining momentum both in clinical and technical use cases. Its usage is reported in oncology [1,2,3], for example, the neoplastic tissues show an impedance spectrum in the high frequency region (>10 MHz) different from that of normal tissue [1]. In cardiology, it not only enables continuous monitoring of the heart action and respiration [4], but also allows for monitoring of heart failure [5]. In neurology, it provides an affordable method to diagnose brain edema and hematoma [6] or monitor secondary brain injury [7]. Due to its sensitivity to blood coagulation [8], thrombus formation [9], blood hematocrit, sedimentation, and dielectric properties [10], it may soon prove useful in various biological scenarios, where mostly biochemistry has been used so far [11,12]. In order to fully develop the diagnostic power of impedance in biological, clinical, and technical settings, the underlying phenomena have to be deeply understood, in order to provide a faithful interpretation. In the current paper, we concentrate on biological samples, as despite rich evidence [13,14], their analysis is still cumbersome, due to technical problems, e.g., with skin contact and other co-factors [15,16] (For example, typically, more than one piece of equipment is used—c.f. [13]), but also due to insufficient understanding of the phenomena, which leads to misinterpretation. A striking example is the distinction between conductance and capacitance. The remark by von Hippel, that the former captures all the phenomena related to energy dissipation [17], including, i.a., dipole rotation in a viscous medium, is completely forgotten.

### 1.1. Biological Samples in an Electric Field

The mechanisms behind the electrical impedance of biological tissues have not yet been fully identified, which limits the ability to interpret the observed phenomena [18]. Currently, the language used to describe changes in tissues during stimulation with external potential typically refers to the theory designed for solids, and not to real dielectrics with losses such as biological tissues [19,20].

Biological samples exhibit a variety of responses to the electric field: from global and local conductance currents related to the physical displacement of ions to the displacement currents of capacitive nature related to such phenomena as atomic orbital deformation, ordering of polar solvents, and surface effects. The latter originates from the change of the width of the double layer, which appears in the area where the potential gradient is nonzero, as only such a field may alter the local concentration of dipoles [17].

Biological tissues may be considered a set of mesoscopic lipid–protein structures filled with an electrolyte. The range of responses of electric polarization is extremely large: from the fastest changes related to deformation of orbitals, located at the optical frequencies, to the slowest changes related to the rotation of large proteins and other polymers, which can be located at millihertz and even below [21,22]. The ordered motion of ions wrapped in hydration shells and accompanied by Brownian motion is relatively slow. The conductance current is ionic in nature and also depends on all factors affecting the diffusion and drift of ions, such as temperature, viscosity coefficient, electrolyte concentration in the tissue, or tortuosity, which affects the effective diffusion coefficient [23]. This physical reality differs from the idealistic picture typically considered in solids, when the current density *j* is in phase with the applied electric field, as governed by the microscopic Ohm law: j=σE. On the contrary, the dispersive character of polarization is expressed in its popular formulation:

(1) $$D\left(t\right)=\varepsilon_{0}E\left(t\right)+\int_{-\infty }^{t}\chi \left(t-\tau \right)\;E\left(\tau \right)\;d\tau$$

where:

- D(t)—electric displacement at time *t*,
- $\varepsilon_{0}$—dielectric permittivity of the vacuum,
- E(t)—electric field intensity at time *t*,
- χ(t−τ)—susceptibility (kernel of the integral).

Therefore, for a comprehensive characterization of conductivity and polarization phenomena in biological samples, it is necessary to develop a method of describing conductivity in the extracellular space and in the cytosol. A theoretical model is proposed below, in Section 3.

**Figure 1:**
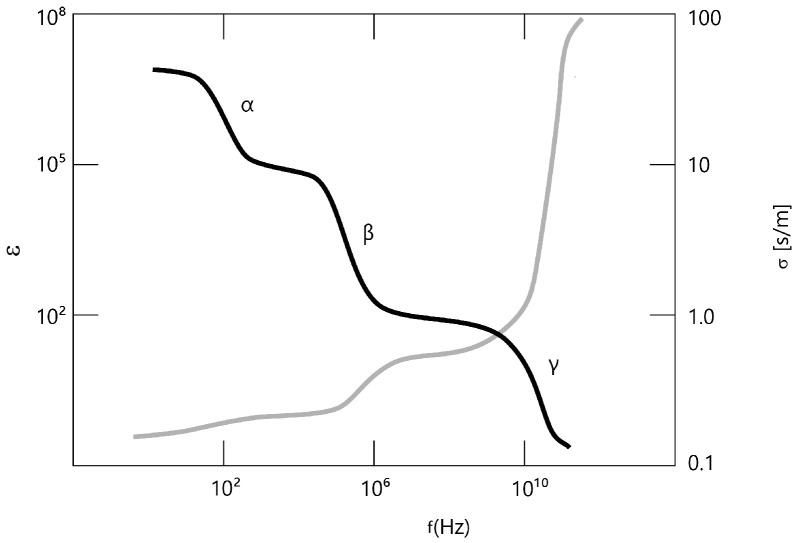
Frequency spectrum of the relative permittivity (black line) and conductivity (grey line), based on [25].

The passive dielectric properties of biological tissue are two material constants: conductivity and permittivity. The conductivity σ (related to the real part of the reciprocal of the electrical impedance) is a quantity used to indicate the ease with which free charges move through a medium (However, the already cited opinion expressed by von Hippel should be considered). In the microscopic approach, it is directly related to charge drift velocity. The permittivity ε (related to susceptance—susceptibility, i.e., the imaginary part of the reciprocal of impedance) is the ability of enclosed charges to polarize inside the medium. Their values depend on frequency. The relationship between frequency and the conductivity and relative permittivity of muscle tissue is shown in Figure 1. The step-wise changes in the spectrum of conductivity and permittivity at certain frequencies are the so-called dispersions, traditionally denoted by Greek letters [24].

### 1.2. Hierarchy of Dispersions

The notation that defines the hierarchy of dispersions was introduced by Schwan [24,25], who postulated the existence of three dispersion mechanisms (α, β, γ) responsible for the structure of the spectra. According to Schwan, the α-dispersion is due to surface admittance and is more sensitive to age and environmental factors than the β-dispersion, which, in turn, was found to depend on the cellular structure and on low-frequency properties caused by cell membranes. It must be noted that the mechanisms behind dispersion are often unclear or unknown [18]. Even the location of the dispersion in a specific frequency range may be much less clear than in Figure 1.

The exact dispersion types are explained below:

- α-dispersion—the window of this dispersion dominates at low frequencies, ranging from a few Hz to several kHz. The mechanisms contributing to this dispersion window are unclear up to now [18]. Three well-known mechanisms are the influence of the endoplasmic reticulum, channel proteins inside the cell membrane, affecting the conductivity, and the relaxation of counter ions on the charged cell surface. This dispersion is most altered by the biological death of the tissue [18].
- β-dispersion—the plasma membrane is a major contributor to this dispersion window due to its capacitive properties. The membranes of the organelles inside the cell also contribute to this phenomenon. The window varies from a few to several hundred kHz. It has been found to heavily depend on cell size. Moreover, for anisotropic tissues, it also depends on the orientation of the sample. Interpretation of this dispersion will be discussed further in the text.
- γ-dispersion—it is caused by water molecules in the intra- and extracellular medium. The dielectric properties of biological tissue are determined by water molecules that have a relaxation frequency at 20 GHz. However, the tissue water contains proteins and other components that broaden a dispersion window, which occupies a wide band in the spectrum, from hundreds of MHz to several GHz. Still, this dispersion correlates with the hydration of the tissue [5,26].

In order to better understand the nature of dispersions, we decided to combine two approaches: a direct measurement of a simple biological model, supported by a numerical study. Both approaches are explained below.

## 2. Experimental Method

There are many different methods for measuring electrical impedance spectra, such as single frequency measurement and electrical bioimpedance spectroscopy [2,3], two- and four-electrode measurements [27], and the bridge method supported by the use of the Cole impedance model [28]. This work focuses on the application of impedance spectroscopy, with a wide range of frequencies.

### Electrochemical Impedance Spectroscopy

Electrochemical Impedance Spectroscopy (EIS) is widely used in chemistry for electrochemical and corrosive research because it enables the characterization of even extremely complex systems [29]. This easy-to-use technique is characterized by the possibility of obtaining rich information [30]; however, it is not always easy to interpret the results [31] (There are numerous problems: the system is not LTI—linear time independent, there exist nonlinearities and temperature drifts, e.g., due to a constant current, that may mimic phase shift [32] etc.) The advantage of using the EIS method is the ability to test separate elements of the electrochemical system, such as charge transfer reactions, diffusion, electrolyte resistance, or geometric sample capacity. It is also possible to test the conductivity of the sample as well as its surface. For the testing of biological samples, the EIS is particularly useful when the same samples are measured in different states (e.g., freshness of meat [33]) and at different temperatures (freezing and thawing process), as in our case.

Biological tissues contain an electrolyte, which makes it possible to treat the tested samples similarly to any electrochemical system. The equilibrium of such a system may be disturbed by applying an AC signal with a given potential and frequency. The response of the system is expressed as a current of amplitude reduced by the sample resistance and phase shifted with respect to the exciting signal. The set amplitude is small to avoid large sample distortions and temperature drift.

Different processes in the sample, e.g., ion transition, electrolyte diffusion, charge transfer, have different time constants. This means that some processes require a shorter time for the system to return to equilibrium after the external stimulus. The existence of multiple time constants affects the frequency dependence of the system response. As a result, most of the processes can be separated sufficiently to allow their identification. Recall that it should be assumed that the condition of the sample during the measurement is constant in time, with no charge/dipole accumulation or temperature drift (the LTI assumption).

In this paper, the Bode diagram is used to present impedance results. It consists of two curves: one in the log (f) – log (Z) coordinates, the other in the log (f) – log (ϕ) coordinates. The Bode diagram makes it easy to determine the number of elements and the frequency range at which they occur.

**Figure 2:**
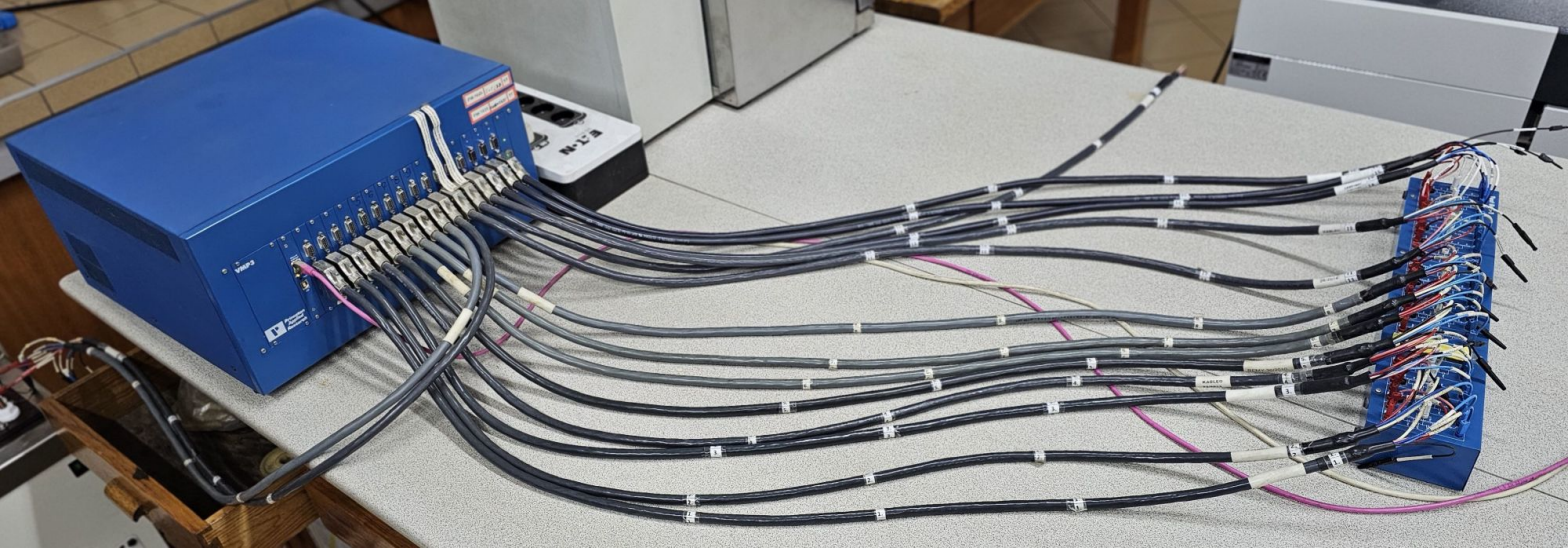
EIS apparatus VMP3 located in the Laboratory of Inorganic Chemistry, Warsaw University of Technology, used for impedance spectroscopy.

The VMP3 EIS apparatus (Figure 2) was used to measure the impedance spectra of biological samples. Impedance spectroscopy was used in the research, obtained with the use of the FRA (Frequency Response Analyzer) function. The signal amplitude for each measurement was 10 mV and the frequency measurements ranged from 100 mHz to 500 kHz. The distribution of the measuring points was logarithmic, with an equal interval between the decades of frequency. Therefore, in the scope in which the research was conducted, 67 measurement points were obtained (10 points per decade). The equipment has 16 channels, which made it possible to perform measurements for 16 samples simultaneously.

The results of impedance spectroscopy (impedance spectra) could be analyzed directly in the measurement software EC-Lab V11.12. This software allows setting of the desired measurement frequencies, as well as displaying of several measurements on one graph, which facilitates the comparison of the results of different samples.

Copper rods were used to perform impedance measurements on field cucumbers during freezing and thawing. The electrodes were placed in the sample so that they were in contact with the tissue. Dry ice was used for freezing. The measurement was performed continuously until the internal temperature was stabilized and the sample was completely frozen. The cucumber was placed in a plastic foil to avoid both direct contact of the sample with dry ice and wetting of the electrodes, which could introduce the electrophoretic effects and sample fluid transfer.

**Figure 3:**
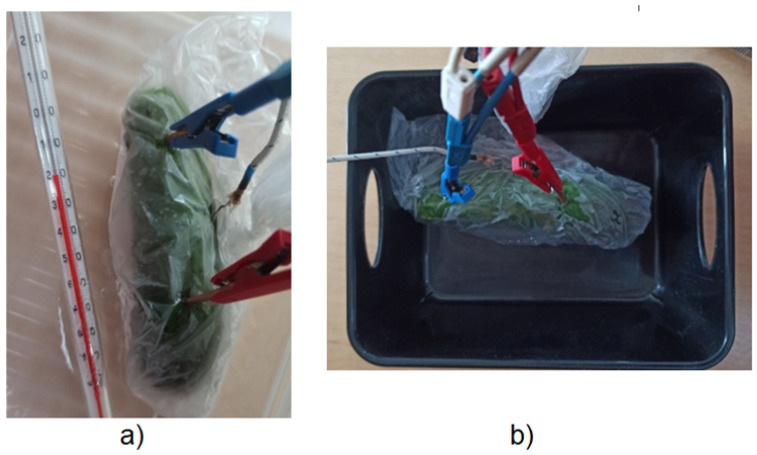
(**a**) Cucumber placed in acetone lowered to the temperature of −20 °C, (**b**) Cucumber placed in a basket to defrost and warm to room temperature.

The cucumber was immersed in acetone, the temperature of which was −28 °C (c.f. Figure 3a). Dry ice was used to maintain a constant temperature of the liquid. As with freezing, the thawing process was also monitored by measuring the impedance spectrum in the loop until a constant internal temperature was reached (the room temperature). To speed up the heating process, the cucumber was taken out of the bath in acetone and dry ice solution, and then placed in a dry basket (Figure 3b).

The field cucumber measurement was used to observe the change in the internal temperature of the tissue during freezing and heating to room temperature. Two alcohol thermometers were used to measure the temperature change inside the sample. One of them was placed in acetone to maintain a constant bath temperature, the other was placed inside the cucumber, between the measuring electrodes, as shown in Figure 3. Both thermometers had a measuring range from −100 to 100 °C, with an accuracy of 1 °C.

Temperature measurement uncertainty was calculated from the formula, which is a type B uncertainty. It contains two terms: the first one relates to the calibration uncertainty related to the graduation on the used alcohol thermometer placed inside the sample, which corresponds to the accuracy of 1 °C, and the second one relates to the experimenter uncertainty, the value of which was assumed to be 0.5°C.

(2) $$u\left(x\right)=\sqrt{\frac{{\left(\Delta x\right)}^{2}}{3}+\frac{{\left(\Delta x_{e}\right)}^{2}}{3}}$$

The resulting measurement uncertainty was 0.65 °C.

**Figure 4:**
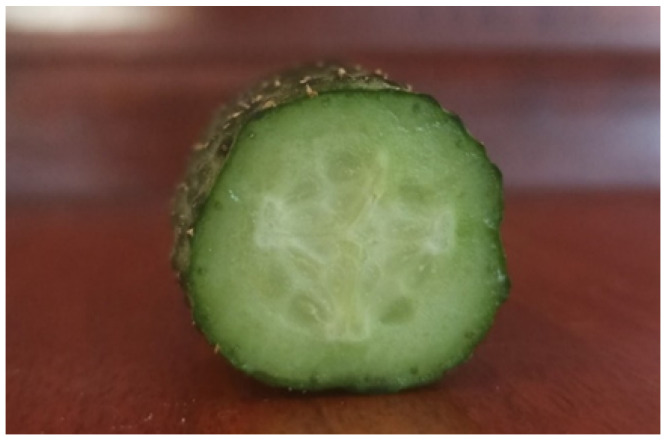
Cross-section of a field cucumber.

Figure 4 shows a cross-section of a field cucumber. It should be noted that it consists mainly of flesh, almost invisible seeds, and a thin skin. This means that lowering the temperature mainly affects the flesh, the majority of which is composed of water, held in position by tiny cellular walls. During the defrosting process, the flesh begins to increase in volume, and as a result, the cellular walls perforate. This phenomenon becomes apparent only after defrosting, as before melting, the structure of the tissue is more or less intact.

The above analysis was repeated for 21 different fruit and vegetable samples and for different positions of the measurement electrodes. We do not report this data, as the results were similar to those presented below, although the effect on the cucumber was the most profound.

## 3. Theoretical Analysis

It is important to note that in the EIS analysis, the analyzed spectrum is decomposed into individual contributions of various mechanisms, which are either resistance-like or capacitance-like. There also exist specific cases, e.g., Warburg impedance, representing the mass transfer process, or constant phase element (CPE), that are not decomposed into subcomponents.

In fact, this analysis can be improved once we depart from the language of Maxwell material constants and equivalent circuits to the underlying physical processes. This actually follows a word of caution by Kenneth Cole himself, who wrote in 1941:

*The numerical values and the configuration of the circuit elements may then be used to describe molecular structure on the one hand and physiological function on the other. But it must be emphasized again that any particular circuit is not necessarily unique and should not be interpreted intuitively. (…) This circuit makes many concessions to simplicity, and several of the obvious defects will be mentioned later* [34].

We define a 2D computational domain in the form of a rectangle, initially filled with an equally spaced amount of ions and coions, of equal concentration. According to Schwan’s nomenclature presented in Section 1.1, the computational domain may represent the interior of a single cell, and its polarization will be relevant to β-dispersion. The external electric field applied to the sample (we have presented elsewhere that such an approach leads to correct results of field calculations [35]; such an approach alleviates other interesting clinical methods [36]) acts on all ions and coions in the finite volume and changes their density, as the ions start to move under the applied field, which subsequently alters the electric potential of the cell. The asymptotic state of a response of the charges to an applied field is reached after a certain time, during which the system is in a transitive state. If the rate of change of the external potential is too high, the polarization in effect does not “catch up”, and the potential gradient between the opposite sides of the cell is reduced and shifted in phase. Such a model of a living cell is simplistic, as it does not consider the presence of an internal structure or any surface effects related to membrane potentials that contribute to the equilibrium [37]. If we consider the ionic displacements as the mechanism for cell polarization, it is reasonable to make an assumption that each given type of cell has a corresponding specific time $\tau_{\beta }$ required for the complete polarization of the cell. This time depends on the size of the cell (its average size over the studied tissue sample, considering also its anisotropy) and the average speed of the ions displacement (drift) process under the influence of the external electric field. The inverse of the $\tau_{\beta }$ (the shortest time needed for a complete tissue polarization) is the dispersion frequency.

In a previous work, it was shown that Debye–Hückel theory can be used to reproduce the classical experimental results in tissue conductance [38]. Current theory goes a few steps further, as in order to calculate the response to a harmonic forcing, the quasistatic approach is insufficient. In physical reality, the system has insufficient time for relaxation, which is a direct reason for the observed dispersion of capacitance and conductance [13], that ranges over many decades (further details may be found in Buchner’s monograph [39]).

We start our analysis from the Poisson equation, so the model is from the first principles. The details of the derivation are given in Appendix A. The result of this derivation is the electrodiffusive model (3). This equation allows us to determine the spatiotemporal distribution of electric potential Ψ at given parameter κ, which resembles the Debye length, known in electrochemistry, and mostly depends on the density of the charge, that may polarize the studied volume (domain). The equation of this type may be considered a bridge between the stationary Poisson–Boltzmann equation (which may be obtained by setting the left-hand side of (3) to zero) and the classical diffusion equation (c.f., e.g., Pietak et al. [40]). The equation used by Pietak et al. [40] contains separate terms representing diffusion and convection. Instead of them, (3) shows only the end result: the nonlinear term with the hyperbolic sine function.

(3) $$\frac{\partial \psi (x,y,t)}{\partial t}=\nabla^{2}\psi \left(x,y,t\right)-\kappa^{2}\sinh\;\left(\psi (x,y,t)\right)+\;w\left(x\right)\;s\left(t\right),$$

The additional terms on the right-hand side of (3) represent the external forcing, applied at the spatial loci defined by the weight function w(x) and varied in time according to s(t) with amplitude *A*. This external forcing, modulated by the spatial weight function, attains its maximum at the left edge of the domain and decays logarithmically along the length of the domain (instead of Dirac delta in *x* direction, we used a version smoothed by a log function to improve the numerical convergence) and the temporal dynamics is harmonic. The frequency of the function s(t) corresponds to the frequency of the external field stimulating the cell. The EIS response is calculated as the amplitude on the right edge of the computational domain, as it reflects the end result of the polarization process. A normalization condition is applied at each numerical step in order to ensure constant charge density. The explicit Euler solver was used, which assured stability up to $10^{2}\;\mathrm{Hz}$, which was sufficient for our purposes. The simulations were performed for various time step sizes, and the results remained stable across all tested values, indicating numerical stability of the integration scheme. All the boundary conditions were Neumann, the Jacobian at edges was calculated using immersed boundary conditions (value of the potential at points outside the border was approximated by the values at border points). The initial conditions were taken from an IID random generator in the range [−0.05, +0.05]. The algorithm was implemented in Python v.3.14.3 and Julia v.1.12.5.

### A Mystery of Alpha Dispersion

It has been clearly shown [24], that, for each tissue under study, the β-dispersion strongly depends on the characteristic lengths of the cells that constitute a certain tissue, and their orientation with respect to the direction of applied current. We propose a hypothesis that, assuming the β-dispersion is related to the polarization of the cell interior, the α-dispersion may be interpreted analogously. The α-dispersion is characterized by the lowest frequencies, which means that it has the longest time constants. Due to the greater sensitivity to environmental factors, such as post-mortem changes of impedance, we may advance a hypothesis that this dispersion may be closely related to the characteristic dimension of the extracellular space, which represents a porous medium (this has already been discussed by Eisenberg [23]; however, he took a completely different modeling approach). The effective diffusion coefficient in such a medium is lower, as the diffusion path is longer than the geometric distance between the starting point and the end point. This is described by tortuosity, which means that a lower frequency corresponds to a longer diffusion path [41]. Diffusion is actually used in physics to measure the porosity of a medium on a microscopic scale, where tortuosity, volume fraction, and the diffusion coefficient are the characteristic parameters [42]. The time required to fully polarize such a medium must be substantially longer than $\tau_{\beta }$, as the typical anatomical distance between structures that naturally limit the extracellular space is macroscopic.

## 4. Results

The description of the results is split into two parts: the experimental results are reported and discussed first, to set the scene for the numerical experiment, which is reported afterwards.

### 4.1. Experimental Results

**Figure 5:**
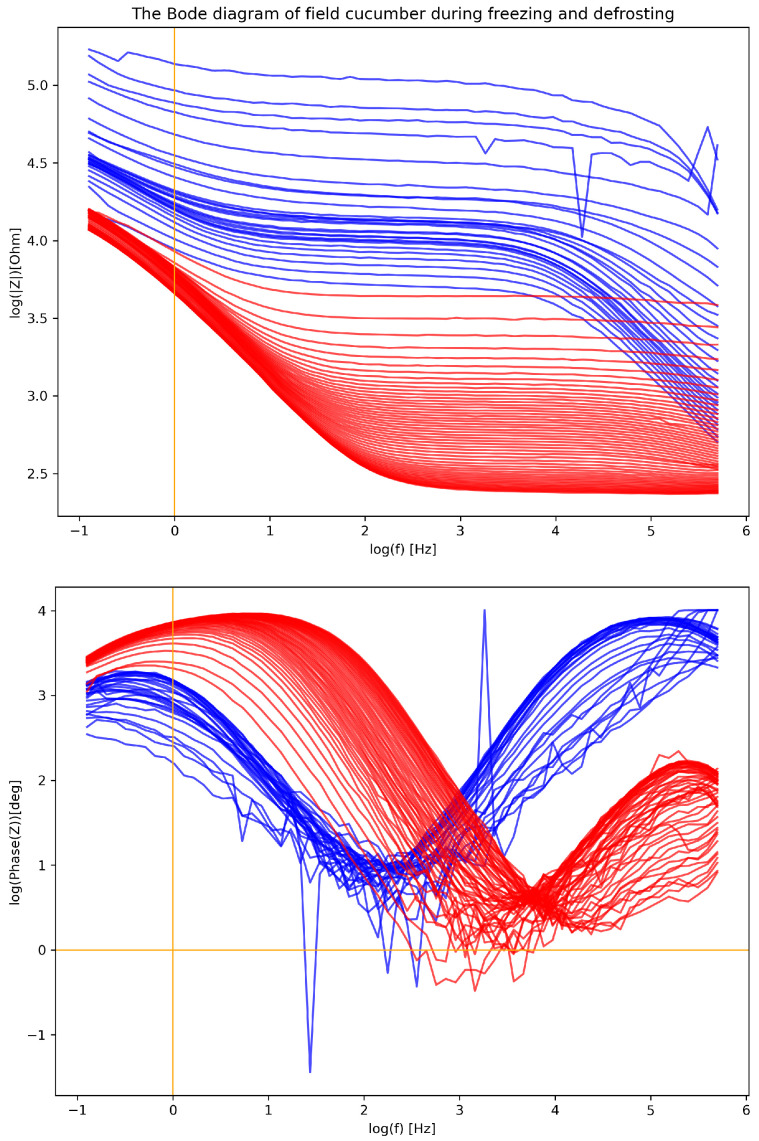
The Bode diagram of the field cucumber during freezing from 22 °C (blue lines) and defrosting from −28 °C (red lines). Freezing has started from bottommost blue line, while defrosting has started from the topmost red line. The initial state is the lowest blue line in the upper panel, whereas the final state is the lowest red line in the upper panel. Lower panel shows the phase of impedance. The initial and the final state for the phase are easy to identify as the traces of the highest concentration. The traces with high noise correspond to the transfer of the sample from the acetone solution to the basket.

Figure 5 shows the results of continuous measurement of the freezing and thawing process from room temperature of 22 °C to −28 °C, which was the temperature of the acetone bath, and back to the room temperature. The jagged signal for certain traces is the result of the relative motion of the test leads while the device was in operation.

During freezing, the impedance amplitude shown in the upper panel of Figure 5 flattens and increases, and the phase curve shown in the lower panel of Figure 5 is relatively constant. For the initial state, the α-dispersion at approx. 1 mHz and β-dispersion at approx. 3.5 kHz are clearly visible, while after thawing, the α-dispersion completely disappears, leaving only a widened β-dispersion shifted towards lower frequencies. In the case of defrosting, the phase spectrum changes significantly. You can see an increase in the phase of impedance at low frequencies with a increase in the internal temperature of the cucumber, as well as the disappearance of dispersion during the heating process.

### 4.2. Discussion of Experimental Results

The first effect to be discussed is the temperature dependence of the spectrum. The observed area in our study covers the spectral range of 100 mHz to 500 kHz, which, according to Schwan’s nomenclature [25], is the area of α- and β-dispersion.

An experiment using the lowering of the sample temperature showed how complex the nature of the electrical impedance spectrum is. When the temperature drops, differences in the propagation properties of the examined tissue are observed. Living tissue is characterized by the presence of an electrolyte (a viscous medium) and the presence of tissue structures (mesoscopic) in which diffusion and drift phenomena may occur. As a result of lowering the sample temperature, the viscosity markedly increases, the rate of both the diffusion and the ordered drift is reduced, and thus the rate of ion transfer is lower, which effectively reduces the drift current. The impeded drift increases the impedance and shifts the dispersion frequency in the spectrum towards higher frequencies. Note that, although the diffusion rate drops with temperature, the Brownian motion of the solvent is diminished as well. We have two contradictory effects. This may explain why the equilibrium is reached faster, as marked by the increase of β-dispersion frequency, equivalent to a decrease in $\tau_{\beta }$. The above explanation is speculative, but it seems valuable to open this discussion on a wider literature basis. Note that below zero, the electrolyte is frozen, and, in ice, the contribution of proton transfer in this solid electrolyte may also be expected [43]. A detailed analysis of the phenomena may be performed using the kinetic approach described, e.g., by Barthel et al. [44].

It is important to understand the α- and β-dispersion discussed in Section 1.1. It is suspected that the α-dispersion is associated with the surface and volume effects of the tissue, while the β-dispersion is associated with the presence of cells and their size [25]. According to the theory, presented in Section 3, the β-dispersion is related to the polarization of the interior of cells, and, more precisely, to the characteristic dimension of the cell.

It is therefore intuitive to assume that the α-dispersion should be related to the characteristic dimension of the extracellular space (porous medium). This means that the effective diffusion coefficient is lower because the path development is longer than the geometrical distance between two points, which is described by the tortuosity coefficient. The diffusion coefficient is used to measure the microscopic structure of a porous medium, and its characteristic parameters are the effective diffusion coefficient and tortuosity [41].

After defrosting the test sample, the frequency of the α-dispersion disappears, while the β-dispersion becomes wider. Before the temperature drop, the tissue structures had a specific time $\tau_{\beta }$ needed for the polarization of the cell and the extracellular space. If the frequency of the β-dispersion has decreased after thawing, it means that the $\tau_{\beta }$ time has increased. The chemical composition and external temperature did not change during the experiment, suggesting that the speed of the ion transfer process did change.

The sample we selected was a field cucumber, as we had expected that it would not tolerate the freezing process. Indeed, after defrosting, significant changes in both the module and phase of the impedance spectra may be encountered. Therefore, it can be concluded that during the thawing process, the cell structure of the tested sample is destroyed, because the β-dispersion, which is associated with the cell size, is shifted downwards as free mass exchange between the extracellular fluid and the cytosol due to membrane perforation becomes possible and the effective length of the cell is increased. The α-dispersion disappears, which resembles the situation after cell death. It is probable that the density of the extracellular fluid rises to such a value that the transport is strongly limited.

### 4.3. Theoretical Results

Two series of numerical experiments were performed. The first sweep was for a given size of the computational domain N=(50,20) and a series of κ∈[0.001,…,0.005] in order to verify if the amplitude of the potential response to the applied field decreases with increasing κ.

**Figure 6:**
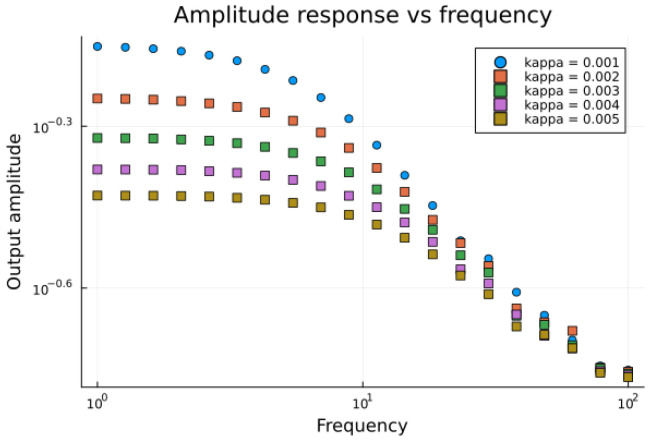
Theoretical spectra for a series of κ∈[0.001,…,0.005] in a log-log plot. Frequency range: f∈ [1,…,100] Hz.

As expected, the plot in Figure 6 shows that increasing the κ—its inverse is a Debye length equivalent reduces the amplitude of the response, which corresponds to an increase in the diffusion impedance.

The aim of the second sweep was to show the main result of the theoretical part: that the increase in the horizontal extent of the computational domain shifts the beta dispersion towards lower frequencies. (Note that we do not consider extracellular space in calculations, this remains as an interesting future direction of development, as basically any mesoscopic structure can be introduced into the computational domain. The only requirement that is substantially different from other computational approaches is that, as the mesoscopic barriers are impermeable, it imposes a limitation on the spatial distribution of the potential Ψ, as within each subdomain the total charge density must be conserved.)

**Figure 7:**
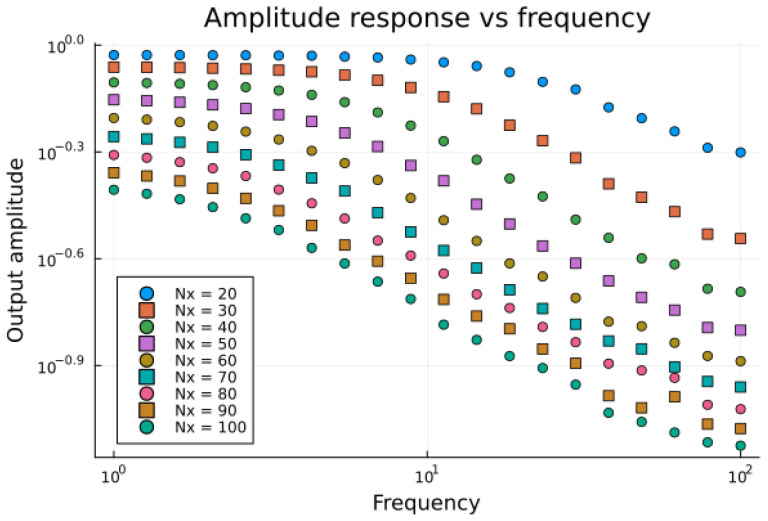
Theoretical spectra for series of $N_{x}\in \left[0.001,\ldots ,0.005\right]$ in a log log plot. Frequency range: f∈ [1,…,100] Hz.

The results of this sweep are depicted in Figure 7. It can be seen that the response amplitude departs from the constant value, and the position of this departure point moves towards lower frequencies as the size of the domain increases.

### 4.4. Discussion of Theoretical Results

It can be seen that the response to the external electric field applied to the computational domain, which represents a single cell of a cucumber, is qualitatively comparable to the experimental results shown in Figure 5. The dependence on κ mimics the dependence on the temperature. The results show that the increase of κ that might have been caused by the temperature drop, or by many other factors that affect the value of κ, leads to the decrease of the amplitude, which is equivalent to an increase in the sample impedance.

### 4.5. Discussion of Model Calibration

In order to be able to directly compare the model with the experimental data, its prior calibration would be necessary. This calibration might be based on:

1. The value of $\tau_{\beta }$ in the intact sample, combined with the optical microscope imaging analysis of the biological sample to determine the size of cells and their distribution.
2. The analysis of the ionic composition of the cytosol, the optical limit of relative dielectric permittivity to obtain a realistic approximation of the ε that appears in the definition of κ.
3. The analysis of ionic strength of the electrolyte, i.e., after homogenization of a sample—e.g., using field-flow fractionation [45].
4. The analysis of the temperature dependence of the low-frequency limit. Note that in this model the diffusion constant *D* practically sets the time scale of numerical units—we haven’t introduced it literally, but in electrodiffusive models, the right-hand side of the equation is multiplied by *D*.
5. The analysis of the response of $\tau_{\beta }$ to changing Nx—the cell length—might be used to determine the spatial scale in μm per numerical grid step.

Note that calibration would require a more realistic theoretical setup, including the presence of the mesoscopic structure, so that the domain structure would mimic the microscopic structure of the cells of the cucumber. Note also that the length of the cell strongly depends on the age of the whole fruit [46]. Accounting for this fact would require either a precise control of the sample age or verification post hoc and ex ante via microscopy, as the literature value of 55 μm is only a crude approximation. Other approaches to calibration can also be used, as the impedance of cell suspensions may also be determined [19,25]. Specifically, blood cells are known to respond vividly to many environmental factors [47], which opens the possibility to develop the biological model and the numerical model side by side in order to improve their quality, to provide added value to future blood studies.

## 5. Conclusions

We have demonstrated the application of the electric impedance spectrum (EIS) to a biological sample. Despite the simplicity of the experimental protocol, the obtained results were rich: they demonstrated a gradual increase in impedance with falling temperature. During the freezing process, the cellular structure of the cucumber tissue was destroyed, which was associated with the “spilling” of ions between individual cells and to the extracellular space. This increased the path of passage of charges, and in consequence, the time required for full tissue polarization also increased. This, in fact, constitutes the model of β-dispersion.

In parallel, we have developed a non-stationary theoretical model, which combines the Poisson–Boltzmann approach with that of the diffusion equation, which allows us to qualitatively mimic the results of the experimental study. The procedure for calibration of the model in time and space was discussed. We have demonstrated, with the help of the model, that increasing the time needed for the tissue to polarize is associated with a broadening and a shift of the impedance spectrum, and thus the repositioning of the dispersion in the frequency range used in the research. A conjecture concerning the origin of α-dispersion was also proposed. The results of this exploratory work are promising, and both the experiment and the computation open a wide area of development. A few future directions are sketched above. To our knowledge, this is one of the first attempts to bridge the molecular phenomena and the corresponding impedance spectrum. The potential use of this technique extends far beyond the analysis of biological samples or medical diagnosis.

## Data Availability

The original data presented in the study are openly available at https://doi.org/10.5281/zenodo.18749066.

## Appendix A. Derivation of the Dynamical Poisson–Boltzmann Equation

Many models for living tissue conductance (c.f. [35] for details) are based on a quite unrealistic assumption, that the volume through which the electric signal is transmitted contains no net charge, and hence no charge has to be considered at all. Therefore, they depart from the Laplace equation. We decided to formulate the model starting from the Poisson equation.

(A1) $$\nabla^{2}\Psi =-\frac{\rho }{\varepsilon },$$

where Ψ stands for electric potential, ρ represents the potential density, and ε represents the dielectric permittivity of the vacuum, but also all the polarization mechanisms that are faster than the characteristic times of the mechanisms discussed here. In impedance literature, this value is often referred to as $\varepsilon_{\infty }$, as it can be calculated as the high-frequency limit of the spectrum. We assume that the net charge within the domain is equal to zero, but the total charge of ions and coions separately differs from zero. This may be expressed as:

(A2) $$c_{+}+c_{-}=0,$$

where $c_{+}$ and $c_{-}$ represent the density of ions and coions, respectively. Next, we assume that the charge density has to be confined with the potential: we assume that at each point they are related by a stationary Boltzmann distribution. This is an approximation, it should be noted that the problem is not electrostatic, as we allow the charge distribution to change in time and space. According to this assumption, the distribution of ions and coions, with respect to the potential, can be expressed as:

(A3) $$\begin{array}{c} c_{+}=c_{0}exp\left(-\beta e\Psi \right) \end{array}$$

(A4) $$\begin{array}{c} c_{-}=c_{0}exp\left(+\beta e\Psi \right), \end{array}$$

where $c_{0}$ denotes the constant charge density, which can be considered the ionic strength of the electrolyte, Ψ is electrostatic potential, *e* stands for the elementary charge, and $\beta =1/k_{B}T$. If we plug (A4) into (A2), we arrive at a nonlinear relation between the charge distribution and the potential—its linearization leads to the known Debye–Hückel equation (c.f. [48] for in-depth discussion):

(A5) $$\rho =e\left(c_{+}-c_{-}\right)=-2ec_{0}sinh\left(\beta e\Psi \right).$$

The Equation (A5) may be applied to (A1), which leads to the Poisson–Boltzmann equation, which is entirely based on potential Ψ.

(A6) $$\nabla^{2}\Psi =\frac{2ec_{0}}{\varepsilon }sinh\left(\beta e\Psi \right).$$

It is important to remember that although the equation does not directly bind the charge density, all the constraints that limit the physical motion of the charge, e.g., entropic barriers or non-permeable mesoscopic structures, have to be applied to the potential during its calculation to ensure its compliance with these constraints. For instance, the right-hand side of the computational domain will polarize only if the condition for a constant ρ is applied by ensuring that for the whole domain the integral of (A5) holds. This condition has to be verified at each step of computation.

In the final step, we introduce a constant κ defined as:

(A7) $$\kappa =\sqrt{\frac{2e^{2}c_{0}}{\varepsilon k_{B}T}}.$$

Here, we tacitly assume that the product βe can be excluded from the argument of the sinh function. With the help of (A7) we can write the final stationary form of the Poisson–Boltzmann equation as:

(A8) $$\nabla^{2}\Psi =\kappa^{2}sinh\left(\Psi \right),$$

where κ stands for the reciprocal of Debye length.

Now, we assume that the stationary Equation (A8) is satisfied only if the relaxation processes are completed. Hence, it is logical to assume that the difference between the left-hand side and the right-hand side of (A8) is a measure of the local imbalance. It is also logical to assume that this imbalance defines the rate of the relaxation processes, which are fueled by the local departure from the stationary value. Therefore, we consider that the time derivative of the potential is proportional to this local imbalance at each point. In consequence, we get to the non-stationary version of the Equation (A8), which attains the form:

(A9) $$\frac{\delta \Psi }{\delta t}=\kappa^{2}sinh\left(\Psi \right)-\nabla^{2}\Psi$$

Note that the time units are abstract, prior to use, the model has to be calibrated by a suitable adjustment of time unit. This concludes the derivation.
